# Hip Arthroscopy During Female Menstruation: A Retrospective Analysis of Safety and Clinical Outcomes With 24‐Month Follow‐Up

**DOI:** 10.1111/os.70382

**Published:** 2026-07-23

**Authors:** Lin He, Zhi‐Yao Lv, Zhang‐Hua Tong, Qing‐Feng Yin, Ran Ji, Liu‐Pu Yang, Ming‐Yang An, Chun‐Bao Li

**Affiliations:** ^1^ Senior Department of Orthopedics Chinese PLA Hospital Beijing China; ^2^ Medical School of Chinese PLA Beijing China; ^3^ Department of Sports Medicine The Second Qilu Hospital of Shandong University Jinan China

**Keywords:** clinical outcomes, female reproductive health, hip arthroscopy, menstruation, safety

## Abstract

**Objectives:**

To investigate the safety and clinical outcomes of hip arthroscopy performed during menstruation in reproductive‐age female patients, and to determine whether surgery during menstruation increases intraoperative risks or adversely affects postoperative recovery. Currently, clear clinical guidelines regarding the timing of elective orthopedic surgeries relative to the menstrual cycle are lacking, often leading to unnecessary surgical rescheduling. Therefore, this study aimed to provide evidence‐based guidance for perioperative management and optimal surgical scheduling, thereby addressing a critical clinical need.

**Methods:**

This retrospective cohort study included reproductive‐age female patients who underwent hip arthroscopy at the First Medical Center of Chinese PLA General Hospital, the Fourth Medical Center of Chinese PLA General Hospital, and the Department of Sports Medicine at the Second Hospital of Shandong University from December 2018 to September 2023. Patients were categorized into the menstruation group (MP) and the non‐menstruation group (NMP) based on whether they were menstruating on the day of surgery. The NMP group consisted of equally sized ovulatory (OP) and luteal (LP) phases. Perioperative outcomes included: intraoperative blood loss, anesthetic dosage, operative time, hemoglobin (Hb) change (within 24 h postoperatively), prothrombin time (PT), and activated partial thromboplastin time (APTT). Postoperative outcomes included analgesic consumption, duration of analgesic use, time to first unassisted weight‐bearing ambulation, complications, and urinary tract infections. Functional outcomes were assessed at 24‐month follow‐up using the Visual Analog Scale (VAS), Modified Harris Hip Score (mHHS), International Hip Outcome Tool‐12 (iHOT‐12), Hip Outcome Score‐Activities of Daily Living (HOS‐ADL), and Hip Outcome Score‐Sports Specific Subscale (HOS‐SSS).

**Results:**

Of 884 eligible patients, 28 were included in the MP group and 56 matched patients in the NMP group. Operative time, anesthetic use, Hb change, PT, and APTT were comparable between the two groups (all *p* > 0.05). No serious complications or urinary tract infections occurred in either group. At the 24‐month follow‐up, both groups demonstrated significant improvements in VAS, mHHS, iHOT‐12, HOS‐ADL, and HOS‐SSS (all *p* < 0.001), with no statistically significant differences observed between the groups.

**Conclusions:**

Hip arthroscopy during menstruation does not increase intraoperative bleeding, impair coagulation, or delay functional recovery for female patients. Menstruation should not be considered a contraindication when appropriate perioperative management is applied.

## Graphical abstract

Analysis of hip arthroscopy performed during menstruation reveals no significant impact on bleeding or postoperative recovery, suggesting that menstruation is not a contraindication for surgery under proper management.

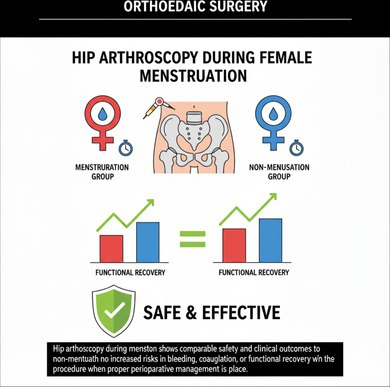

## 1 Introduction

Menstruation is a physiological process regulated by cyclic fluctuations of estrogen and progesterone [1]. During this phase, circulating hormone levels reach their nadir, accompanied by physiological changes such as enhanced fibrinolytic activity and subtle alterations in coagulation factors. Additionally, the decline in these hormones may heighten pain perception, potentially impacting postoperative analgesic requirements and rehabilitation adherence. Historically, these changes have led many surgeons to avoid elective procedures during menstruation due to concerns regarding increased bleeding, heightened pain sensitivity, and delayed recovery. This traditional approach frequently complicates surgical scheduling and healthcare resource utilization, particularly for female patients [2, 3, 4].

Hip arthroscopy has evolved into a mainstream option for managing pathologies such as femoroacetabular impingement (FAI), acetabular labral tears, synovial disorders, and other intra‐articular conditions [5]. The procedure is favored for its minimal soft‐tissue trauma and limited intraoperative bleeding [6]. These benefits make hip arthroscopy particularly favored among younger, active patients [7]. Importantly, approximately two‐thirds of these procedures are performed on women, many of whom are of reproductive age [8]. As the volume of these procedures continues to grow, an increasing number of women inevitably undergo hip arthroscopy during menstruation, raising critical questions regarding the appropriateness of surgical timing.

From a surgical perspective, it is unclear whether menstrual‐associated changes in coagulation could impair intraoperative hemostasis or visualization. Furthermore, menstrual discomfort or mood fluctuations may reduce patient compliance with essential rehabilitation exercises. This uncertainty not only heightens patient anxiety but also results in suboptimal surgical scheduling and inefficient use of healthcare resources [9, 10]. Currently, there are no standardized guidelines to inform the decision‐making process, and the decision often relies on individual surgeon judgment, leading to variability in clinical practice.

Given the lack of systematic research, there is an urgent need to assess whether menstruation significantly affects the safety and clinical outcomes of hip arthroscopy. The primary objective of this study is to systematically evaluate the impact of menstrual status on patients undergoing hip arthroscopy, focusing on three core areas: (i) to investigate intraoperative safety and hematological stability, including blood loss and perioperative coagulation function; (ii) to assess early postoperative recovery, such as analgesic consumption, time to unassisted ambulation, and acute complications; and (iii) to determine long‐term functional outcomes and pain relief at the 24‐month follow‐up. We hypothesize that, with appropriate perioperative management, menstruation will not increase the risks or compromise the short‐term outcomes of hip arthroscopy.

## 2 Methods

### 2.1 Study Design and Patient Selection

This retrospective case–control study was conducted in accordance with the ethical principles of the Declaration of Helsinki and was approved by the Institutional Review Boards of the First Medical Center of Chinese PLA General Hospital, the Fourth Medical Center of Chinese PLA General Hospital, and the Department of Sports Medicine at the Second Hospital of Shandong University (Approval No. 2021KY031‐HS001). Female patients who underwent hip arthroscopy at these three institutions between December 2018 and September 2023 were retrospectively reviewed. Inclusion criteria were: (1) premenopausal women aged 18–45 years; (2) regular menstrual cycles ranging from 26 to 40 days; (3) clear surgical indications for hip arthroscopy, specifically limited to femoroacetabular impingement (FAI) and/or acetabular labral tears; (4) primary hip arthroscopy; and (5) American Society of Anesthesiologists (ASA) physical status classification I–II. Exclusion criteria were: (1) hematological disease, coagulation disorders, or long‐term anticoagulant/antiplatelet use; (2) severe cardiovascular disease, malignancy, autoimmune disease, or endocrine disorders; (3) prior hip arthroplasty, arthrodesis, septic arthritis, or osteomyelitis; (4) advanced hip dysplasia (Crowe type III–IV) or severe osteoporosis; (5) use of hormonal contraceptives or other therapies altering the natural menstrual cycle; and (6) participation in another clinical trial or treatments affecting coagulation within 3 months prior to surgery.

### 2.2 Group Matching

Baseline data, including age, body mass index (BMI), ASA classification, hip disease diagnosis, and menstrual status, were extracted. Menstrual status was determined based on preoperative inquiries to the patients and their medical records. Patients were divided into the menstruation group (MP) and the non‐menstruation group (NMP) according to surgical timing. The NMP group included patients in the ovulatory (OP) and luteal (LP) phases, with equal representation of both phases. To ensure comparability, patients were matched at a 1:2 ratio (MP:NMP) with similar baseline characteristics.

### 2.3 Surgical Technique

All procedures were performed by senior surgeons experienced in hip arthroscopy, following a standardized surgical protocol. Under general anesthesia, patients were positioned supine. The contralateral arm was fixed to an abduction frame, and the operative‐side arm was suspended across the chest. The buttocks were aligned with the distal end of the table, and bony prominences at the ankles were protected with padding before applying traction boots. The contralateral leg was fixed in approximately 45° abduction, while the operative leg was placed in neutral with 15° internal rotation. A padded perineal post‐free traction technique was used, with countertraction achieved by a reinforced strap across the contralateral groin and friction between the body and operating table.

**FIGURE 1:**
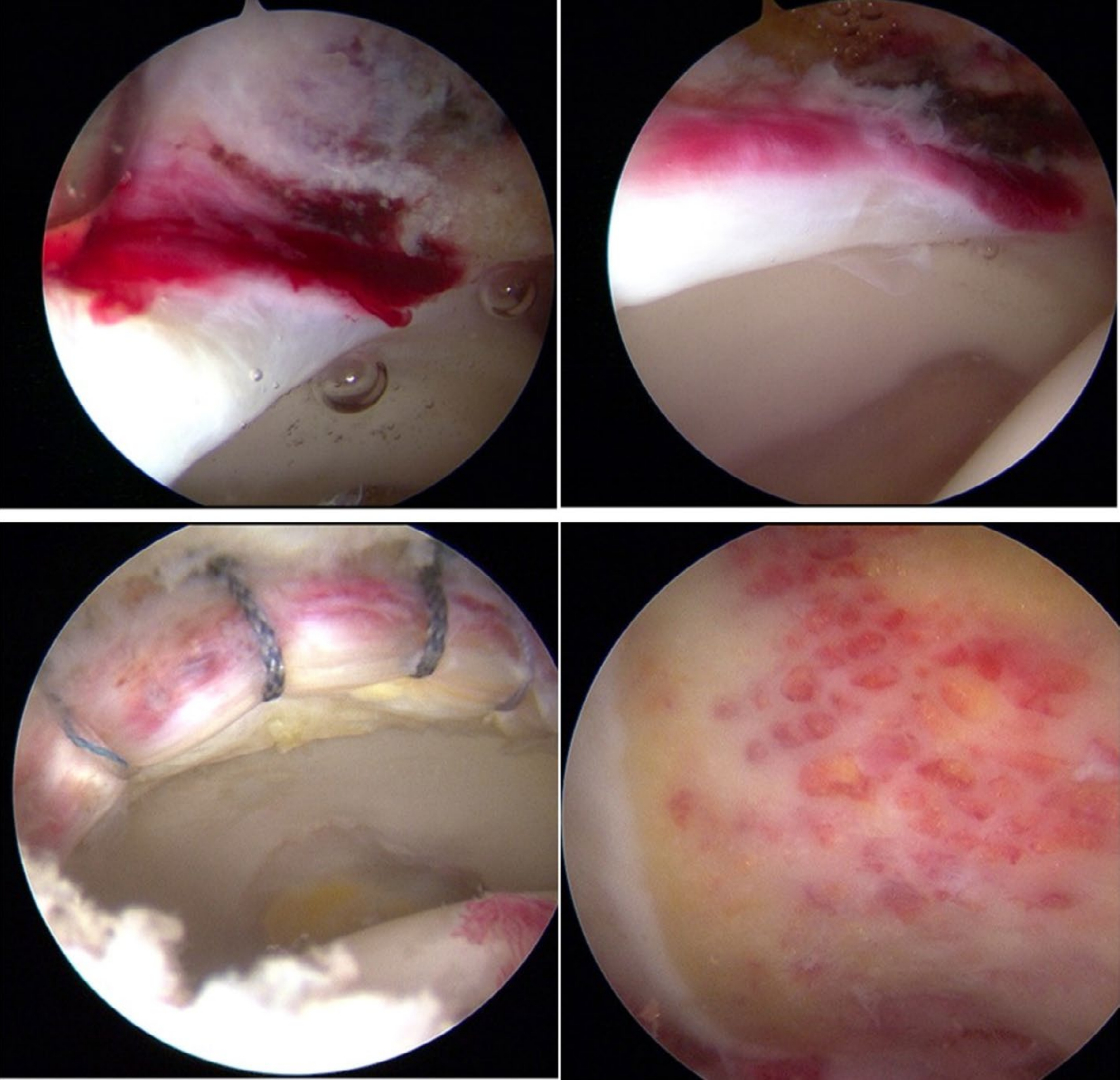
Representative intraoperative arthroscopic views of labral repair.

Traction was gradually applied with the table tilted 10°–15° Trendelenburg until adequate joint distraction (8–10 mm) was achieved without body displacement. Final traction ranged from 10 to 20 kg under fluoroscopic guidance. Standard portals, including anterolateral (AL), mid‐anterior (MA), and modified distal anterolateral accessory (DALA), were established (representative intraoperative images are shown in Figure 1). Central compartment procedures included debridement, acetabular rim trimming, and labral repair. In both groups, all patients (100%) underwent debridement, while 89.3% (25/28 in MP; 50/56 in NMP) underwent acetabular rim trimming, and 78.6% (22/28 in MP; 44/56 in NMP) underwent labral repair. The distribution of surgical techniques was consistent across the matched cohorts. After gradual release of traction, peripheral compartment procedures involved cam resection and capsular closure. At the conclusion, patients were returned to a horizontal supine position.

Postoperative rehabilitation followed a standardized and protocol‐driven program to ensure consistency among patients. Early passive range‐of‐motion exercises were initiated on postoperative day 1, followed by isometric quadriceps and gluteal strengthening within the first week. Progressive weight‐bearing was allowed according to intraoperative findings and patient tolerance. For those who underwent labral repair or extensive osteoplasty, partial weight‐bearing with crutches was maintained for approximately 4–6 weeks before gradual transition to full weight‐bearing. In cases with capsular closure, controlled external rotation and extension were emphasized to prevent overstretching. Return to sports‐specific or high‐load activities was permitted between 3 and 6 months postoperatively, depending on functional recovery and absence of pain.

### 2.4 Outcome Measures

The following data were collected:

- Baseline characteristics: age, height, weight, BMI, age at menarche, menstrual cycle length and duration, disease type, and surgical side.
- Operative variables: anesthetic dosage, intraoperative blood loss, and operative time.
- Laboratory data: hemoglobin (Hb) levels and coagulation parameters (prothrombin time [PT], activated partial thromboplastin time [APTT]) preoperatively and at 24 h postoperatively.
- Perioperative outcomes: cumulative analgesic use and duration, hospital stay, and time to first unassisted ambulation.
- Clinical assessments: hip pain, function, and psychological status evaluated at 1 week preoperatively and at 24 months postoperatively using the Visual Analog Scale (VAS), Modified Harris Hip Score (mHHS), international Hip Outcome Tool‐12 (iHOT‐12), Hip Outcome Score‐Activities of Daily Living (HOS‐ADL), and Hip Outcome Score‐Sport Specific Subscale (HOS‐SSS).
- Complications: postoperative complications within 24 months, including wound infection, urinary tract infection, bleeding, menstruation‐related abdominal pain, deep vein thrombosis, persistent hip pain, and restricted motion.

### 2.5 Statistical Analysis

All analyses were performed using SPSS version 27.0 (IBM, Armonk, NY, USA). Continuous variables were tested for normality and homogeneity of variance. Normally distributed data with equal variance were presented as mean ± standard deviation (SD) and compared using paired‐samples *t*‐tests (within groups) or independent‐samples *t*‐tests (between groups). Non‐normally distributed data and/or unequal variance were expressed as median (interquartile range, IQR) and compared using the Wilcoxon signed‐rank test (within groups) or the Mann–Whitney *U* test (between groups). Categorical variables were expressed as frequencies and percentages, with between‐group comparisons performed using the χ^2^ test or Fisher's exact test when expected frequencies were < 5. A two‐sided *p* value < 0.05 was considered statistically significant.

## 3 Results

### 3.1 Patient Demographics and Baseline Characteristics

**FIGURE 2:**
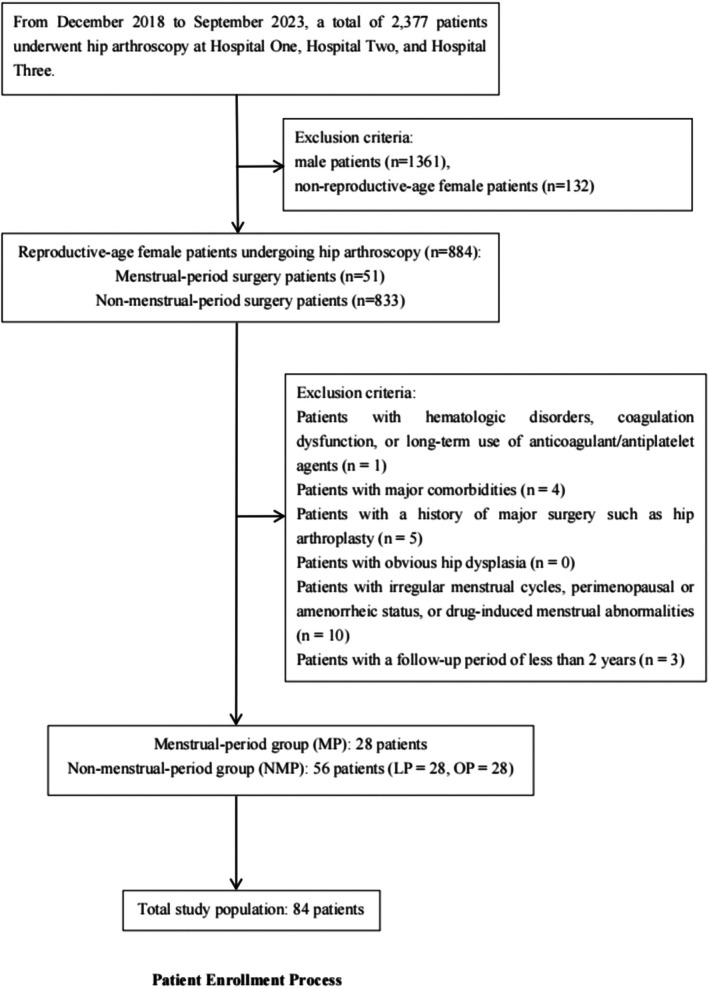
Flow diagram of patient enrollment and inclusion.

**FIGURE 3:**
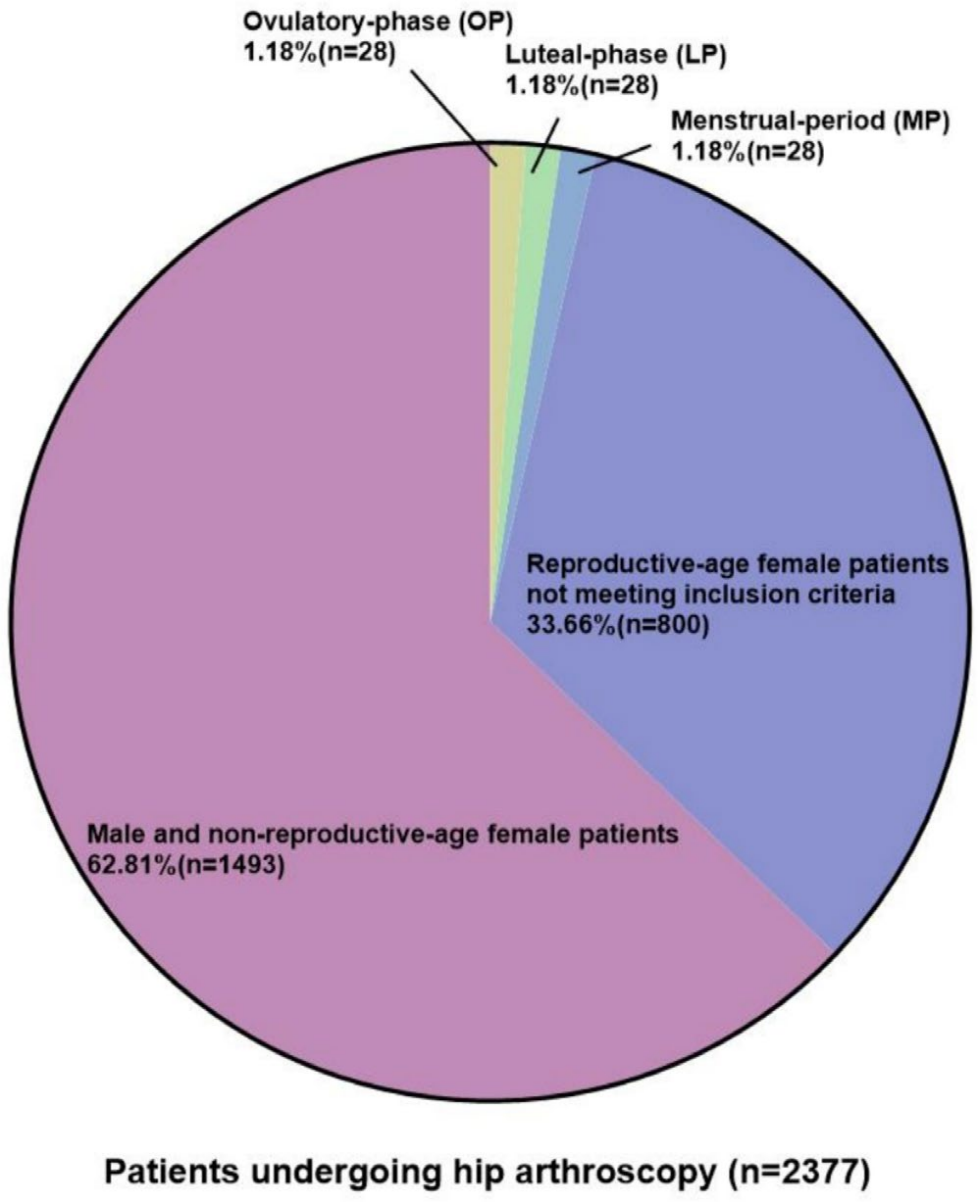
Distribution of patients included in the study.

Between December 2018 and September 2023, a total of 2377 patients underwent hip arthroscopy at the First Medical Center of Chinese PLA General Hospital (*n* = 804, 33.8%), the Fourth Medical Center of Chinese PLA General Hospital (*n* = 1058, 44.5%), and the Second Hospital of Shandong University (*n* = 435, 18.3%). Among them, 884 patients were reproductive‐age women (285 at the First Medical Center, 461 at the Fourth Medical Center, and 138 at the Second Hospital). After applying inclusion and exclusion criteria, 28 patients who underwent surgery during menstruation were included in the MP group (5 from the First Medical Center, 18 from the Fourth Medical Center, and 5 from the Second Hospital; Figures 2 and 3). The NMP group consisted of 56 patients matched at a 1:2 ratio with the MP group according to age, BMI, surgical indication, disease type, surgical side, and procedure, including 28 patients in the luteal phase (LP) and 28 in the ovulatory phase (OP). The distribution across the three centers was consistent with that of the MP group. Baseline characteristics showed no significant differences between groups (all *p* > 0.05), indicating good comparability (Table 1).

**TABLE 1:** Baseline characteristics of patients in the menstrual‐period group and the non‐menstrual‐period group.

Characteristics	Menstrual‐period group (*N* = 28)	Non‐menstrual‐period group (*N* = 56)	*p*
Age at surgery (years), median [IQR]	31.0 [26.3, 41.8]	37.0 [31.0, 42.0]	0.121
Height (cm), mean (SD)	162.86 (5.10)	163.55 (5.43)	0.573
Weight (kg), mean (SD)	61.48 (9.55)	61.34 (9.67)	0.949
BMI (kg/m^2^), median [IQR]	23.0 [19.9, 25.8]	22.5 [20.2, 24.7]	0.772
Surgical side (Left/Right), *n*	14/14	28/28	1.000
Age at menarche (years), median [IQR]	13.0 [13.0, 14.0]	13.0 [12.0, 14.0]	0.864
Menstrual cycle (days), median [IQR]	28.0 [26.0, 30.0]	28.0 [27.0, 30.0]	0.260
Duration of menstruation (days), median [IQR]	5.0 [5.0, 6.0]	5.5 [5.0, 6.8]	0.374

*Note:* Data are presented as mean ± standard deviation or median (interquartile range); *p* value < 0.05 was considered statistically significant.

### 3.2 Perioperative Outcomes

Perioperative outcomes were largely consistent between groups. Intraoperative blood loss was 12.10 [8.2, 13.8] mL in the MP group and 10.65 [8.0, 14.0] mL in the NMP group, with no significant difference (*p* = 0.939). Operative time was 111.89 ± 13.96 min in the MP group and 110.14 ± 15.33 min in the NMP group, also without significant difference (*p* = 0.613). Hemoglobin changes from baseline to 24 h postoperatively were comparable between groups, indicating similar intraoperative blood loss and postoperative hematologic recovery. Coagulation function (PT and APTT) remained within the normal range both preoperatively and 24 h postoperatively in both groups, with no significant differences observed (Table 2). These findings suggest that menstruation did not adversely affect coagulation function or intraoperative hemostasis.

**TABLE 2:** Comparison of perioperative outcomes between the menstrual‐period group and the non‐menstrual‐period group.

Variable	Menstrual‐period group (*N* = 28)	Non‐menstrual‐period group (*N* = 56)	*p*
Hb (g/L), preoperative, mean (SD)	122.54 (13.38)	123.25 (10.15)	0.786
Hb (g/L), postoperative, median [IQR]	118.00 [106.8, 127.8]	116.50 [113.0, 125.8]	0.823
PT (s), preoperative, mean (SD)	11.90 (0.81)	11.84 (0.80)	0.775
PT (s), postoperative, median [IQR]	12.05 [11.5, 12.4]	11.80 [11.5, 12.3]	0.604
APTT (s), preoperative, median [IQR]	30.10 [28.2, 33.3]	29.40 [28.0, 31.3]	0.241
APTT (s), postoperative, median [IQR]	28.40 [27.6, 30.6]	28.90 [27.8, 30.8]	0.459
Intraoperative blood loss (mL), median [IQR]	12.10 [8.2, 13.8]	10.65 [8.0, 14.0]	0.939
Total operative time (min), mean (SD)	111.89 (13.96)	110.14 (15.33)	0.613
Total anesthetic dosage (mg), median [IQR]	28.54 [2.0, 102.1]	2.12 [2.0, 52.0]	0.094
Total analgesic dosage (mg), median [IQR]	800.00 [600.0, 950.0]	800.00 [600.0, 1000.0]	0.467
Duration of analgesic use (days), median [IQR]	3.00 [3.0, 4.0]	3.50 [3.0, 5.0]	0.351
Length of hospital stay (days), median [IQR]	4.00 [4.0, 5.0]	4.50 [4.0, 6.0]	0.377

*Note:* Data are presented as mean ± standard deviation or median (interquartile range); *p* value < 0.05 was considered statistically significant.

### 3.3 Postoperative Complications and Recovery

At 24‐month follow‐up, complication rates were low in both groups. The MP group reported 6 complications (21.4%) and the NMP group 7 (12.2%), with no significant difference (*p* = 0.286; Table 3). Reported complications were primarily mild functional limitations, such as restricted hip motion and incomplete functional recovery. No patients in either group developed urinary tract infection, menstruation‐related abdominal pain, deep vein thrombosis, significant postoperative bleeding, or required reoperation. The time to first unassisted ambulation showed no significant difference between groups (*p* = 0.846; Table 3). Overall, these findings indicate comparable postoperative safety and recovery outcomes between patients who underwent surgery during menstruation and those who did not.

**TABLE 3:** Comparison of postoperative complications and recovery outcomes between the menstrual‐period group and the non‐menstrual‐period group.

Variable	Menstrual‐period group (*N* = 28)	Non‐menstrual‐period group (*N* = 56)	*p*
Postoperative complications, *n* (%)	6 (21.43%)	7 (12.50%)	0.286
First use of assistive tools for weight‐bearing (days), median [IQR]	28.00 [21.0, 28.0]	28.00 [16.30, 29.0]	0.846

*Note:* Data are presented as mean ± standard deviation or median (interquartile range); *p* value < 0.05 was considered statistically significant.

### 3.4 Pain and Functional Outcomes at 24‐Month Follow‐Up

All patients completed a 24‐month follow‐up after surgery. At 24 months postoperatively, both groups demonstrated significant pain relief and functional improvement compared with baseline. Duration of postoperative pain did not differ significantly between the MP and NMP groups. VAS scores were markedly lower at 24 months than preoperatively in both groups, with no between‐group difference (*p* > 0.05). Similarly, functional scores including mHHS, iHOT‐12, HOS‐ADL, and HOS‐SSS all showed significant postoperative improvement in both groups, with no significant differences between the MP and NMP groups at 24‐month follow‐up (all *p* > 0.05; Table 4). These results suggest that hip arthroscopy performed during menstruation does not negatively impact long‐term functional recovery.

**TABLE 4:** Comparison of postoperative pain and functional recovery between the menstrual‐period group and the non‐menstrual‐period group.

Variable	Menstrual‐period group (*N* = 28)	Non‐menstrual‐period group (*N* = 56)	*p*
VAS score, preoperative, median [IQR]	5.00 [0.8, 6.8]	5.00 [5.0, 7.0]	0.134
VAS score, postoperative, median [IQR]	1.00 [0.0, 3.0]	1.00 [0.0, 2.0]	0.830
*p*‐value	< 0.001*	< 0.001*	
mHHS score, preoperative, median [IQR]	57.50 [51.5, 88.0]	60.50 [50.0, 70.8]	0.506
mHHS score, postoperative, median [IQR]	86.00 [78.5, 94.5]	86.00 [82.0, 93.0]	0.849
*p*‐value	< 0.001*	< 0.001*	
iHOT‐12 score, preoperative, median [IQR]	60.50 [37.3, 83.0]	53.00 [43.0, 63.8]	0.271
iHOT‐12 score, postoperative, median [IQR]	89.00 [80.0, 108.8]	93.00 [83.5, 104.0]	0.602
*p*‐value	< 0.001*	< 0.001*	
HOS‐ADL score, preoperative, median [IQR]	36.00 [22.3, 56.8]	35.00 [27.3, 50.8]	0.572
HOS‐ADL score, postoperative, median [IQR]	55.00 [51.0, 61.8]	60.00 [54.0, 64.0]	0.093
*p*‐value	< 0.001*	< 0.001*	
HOS‐SSS score, preoperative, median [IQR]	16.50 [3.3, 24.0]	13.00 [3.3, 19.0]	0.400
HOS‐SSS score, postoperative, median [IQR]	31.50 [22.3, 34.8]	31.50 [26.0, 35.0]	0.521
*p*‐value	< 0.001*	< 0.001*	

*Note:* Data are presented as mean ± standard deviation or median (interquartile range); *p* value < 0.05 was considered statistically significant.

* A statistically significant difference was found for this value.

## 4 Discussion

### 4.1 Main Findings of This Study

Hip arthroscopy has rapidly emerged as a widely adopted minimally invasive procedure for the management of femoroacetabular impingement (FAI), acetabular labral tears, synovial disorders, and other intra‐articular hip pathologies [5]. Over the past decade, its utilization has grown substantially; Zusmanovich et al. reported an 85% increase in procedure incidence between 2011 and 2018, with a progressive rise in the proportion of female patients during this period [11, 12]. Despite this trend, there are currently no clear clinical recommendations addressing whether hip arthroscopy should be performed during menstruation in women of reproductive age. Menstrual physiology is characterized by cyclic fluctuations in estrogen and progesterone, transient changes in coagulation activity, and immune modulation—factors that may theoretically influence perioperative safety and postoperative recovery. Nevertheless, the effect of menstrual status on outcomes following hip arthroscopy remains largely unexplored [12].

To address this knowledge gap, we conducted a multicenter retrospective study with a 24‐month follow‐up to evaluate whether menstruation influences surgical safety or postoperative recovery after hip arthroscopy. Although mean intraoperative blood loss was slightly higher in the menstruating (MP) group compared with the non‐menstruating (NMP) group, the difference was not statistically significant. Hemoglobin, prothrombin time (PT), and activated partial thromboplastin time (APTT) remained stable before and 24 h after surgery, indicating that menstruation did not increase perioperative bleeding risk or impair coagulation function. Because urinary catheterization is often required during surgery in menstruating patients, there has been concern regarding a potential rise in urinary tract infections; however, no patients in the MP group developed urinary symptoms, suggesting no additional infection risk. Complication rates were comparable between groups and were limited to mild functional complaints such as transient motion restriction or incomplete recovery. At 24 months, both groups demonstrated significant improvements in VAS, mHHS, iHOT‐12, HOS‐ADL, and HOS‐SSS scores, with no intergroup differences, indicating that menstruation neither delayed rehabilitation nor compromised long‐term outcomes. Consequently, these findings suggest that when a patient's menstrual cycle coincides with scheduled elective hip arthroscopy, routine postponement may be clinically unnecessary.

### 4.2 Intraoperative Safety and Fluid Perfusion Dynamics

One interesting observation in our study is that the theoretical rise in systemic fibrinolytic activity during menses did not result in increased bleeding or compromised visualization. This may be attributed to the technical nature of hip arthroscopy. Unlike open hip surgery, hip arthroscopy is a minimally invasive technique that involves minimal soft‐tissue dissection, thereby limiting the exposure of vascularized tissue [13]. Furthermore, the procedure relies on continuous fluid irrigation under controlled pressure. This creates a “tamponade effect” that physically compresses small capillaries within the joint capsule, maintaining a clear surgical field despite any subtle systemic physiological variations [14]. These combined mechanical factors likely serve as a protective buffer, effectively counteracting potential menstrual‐related bleeding risks and ensuring both surgical efficiency and patient safety.

### 4.3 Comparison With Existing Research in Other Disciplines

Similar observations have been reported across other surgical disciplines, including plastic, cardiac, gynecologic, and ophthalmologic procedures [8, 15, 16, 17]. In plastic surgery, Findikcioglu et al. [8] analyzed 107 reproductive‐age women undergoing rhinoplasty and noted slightly greater intraoperative bleeding during the ovulatory phase than during menstruation; however, the difference was not clinically meaningful and did not influence postoperative outcomes. In a prospective abdominoplasty study, the same group found no significant differences in blood loss, 24‐h drainage volume, or hemoglobin decrease between menstrual and non‐menstrual patients, and no severe bleeding or major complications were observed [15]. In ophthalmology, Lin et al. [16] evaluated 155 women undergoing vitreoretinal surgery and demonstrated that menstrual status did not significantly affect intraoperative or postoperative bleeding, and regression analysis failed to identify menstruation as an independent risk factor. Even in high‐risk cardiac procedures, Das et al. [17] reported comparable intraoperative blood loss between menstrual and non‐menstrual patients, and menstruation neither disrupted subsequent cycles nor increased perioperative bleeding despite the use of cardiopulmonary bypass and postoperative anticoagulation. Collectively, these findings reinforce the overall safety of performing surgery during menstruation and provide indirect evidence supporting its feasibility in hip arthroscopy. By contextualizing our data with these findings across diverse fields, our study provides the first orthopedic evidence confirming that systemic coagulation profiles and local tissue homeostasis remain stable during menses under arthroscopic visualization.

### 4.4 Clinical Management and Rehabilitation Compliance

Consistent with previous research, our findings demonstrate that menstruation did not adversely affect the safety, recovery, or long‐term outcomes of hip arthroscopy. From a clinical management standpoint, surgery is often postponed when it coincides with menstruation, which may prolong waiting times, increase healthcare utilization, and heighten patient anxiety [17]. Based on our results, we suggest that, after excluding high‐risk conditions such as dysmenorrhea, anemia, or coagulation disorders, hip arthroscopy can be safely performed during menstruation without unnecessary delays. Early mobilization and adherence to standardized rehabilitation protocols remain critical for optimal functional recovery. In this study, all patients followed the same rehabilitation regimen regardless of menstrual status, indicating that appropriate preoperative counseling and structured rehabilitation guidance can effectively alleviate concerns regarding postoperative compliance or potential cyclic discomfort.

### 4.5 Limitations and Strengths of This Study

The primary strength of this study lies in its multicenter retrospective design, which systematically evaluated the effect of menstruation on hip arthroscopy—an area that has not been previously explored in orthopedic research. By integrating data from three high‐volume institutions across different regions and healthcare systems, we reduced the selection bias inherent to single‐center studies and enhanced institutional consistency. Rigorous inclusion and exclusion criteria ensured cohort homogeneity, thereby improving the reliability and external validity of our results. Moreover, the use of multicenter data increased the statistical power and representativeness of our findings, aligning the study with international standards for clinical research.

Despite these strengths, several limitations must be acknowledged. Notably, our final sample size for the MP cohort remained relatively modest, which inherently restricts statistical power and elevates the probability of a type II error. Additionally, because this was a retrospective analysis, baseline serum hormone concentrations were not quantitatively assayed; thus, a direct correlative analysis between granular endocrine fluctuations and parameters like micro‐vascular oozing or acute pain thresholds was not feasible. Furthermore, while a 24‐month follow‐up is standard for establishing medium‐term functional benchmarks, it may be insufficient to fully capture late‐stage joint outcomes, including asymptomatic heterotopic ossification or subsequent conversion to total hip arthroplasty. It is also worth noting that although all procedures were guided by a standardized institutional protocol among senior arthroscopy specialists, subtle inter‐operator technical variations across the three participating centers could introduce minor confounding. Lastly, despite our comprehensive matching of baseline demographics and joint pathologies, residual confounding from unmeasured variables—such as preoperative psychological resilience—cannot be entirely dismissed. Consequently, future prospective trials incorporating larger cohorts and longitudinal hormone profiling remain desirable to further expand upon these preliminary findings.

## 5 Conclusion

Hip arthroscopy during menstruation does not increase intraoperative bleeding, impair coagulation, or delay functional recovery for female patients. Menstruation should not be considered a contraindication when appropriate perioperative management is applied.

## Author Contributions

**Lin He:** writing – original draft, writing – review and editing. **Zhang‐Hua Tong:** writing – review and editing. **Ming‐Yang An:** writing – review and editing. **Chun‐Bao Li:** project administration. **Qing‐Feng Yin:** writing – review and editing. **Ran Ji:** writing – review and editing. **Zhi‐Yao Lv:** writing – review and editing. **Liu‐Pu Yang:** writing – review and editing.

## Funding

The authors have nothing to report.

## Disclosure

We hereby declare that all authors listed in this manuscript meet the authorship criteria according to the latest guidelines of the International Committee of Medical Journal Editors (ICMJE). Additionally, all authors have reviewed and approved the final manuscript and agree with its submission to this journal.

## Ethics Statement

The study was conducted in accordance with the Declaration of Helsinki and was approved by the Ethics Committee of Chinese PLA General Hospital (Approval No. 2021KY031‐HS001). Informed consent was obtained from all individual participants included in the study.

## Conflicts of Interest

The authors (L.H., Z.‐Y.L., Z.‐H.T., Q.‐F.Y., R.J., L.‐P.Y., M.‐Y.A., C.‐B.L.) declare that the research was conducted in the absence of any commercial or financial relationships that could be construed as a potential conflicts of interest.

## Data Availability

The data that support the findings of this study are available from the corresponding author upon reasonable request.
